# Tuberculosis-specific antigen stimulated and unstimulated interferon-γ for tuberculous meningitis diagnosis: A systematic review and meta-analysis

**DOI:** 10.1371/journal.pone.0273834

**Published:** 2022-08-30

**Authors:** Fangyu Shi, Xia Qiu, Mingjing Yu, Yan Huang

**Affiliations:** 1 Department of Health Management Center, West China Hospital, Sichuan University, Chengdu, China; 2 Department of Pediatrics, West China Second University Hospital, Key Laboratory of Obstetric & Gynecologic and Pediatric Diseases and Birth Defects of Ministry of Education, Sichuan University, Chengdu, China; 3 Department of Respiratory and Critical Care Medicine, West China Hospital, Sichuan University, Chengdu, China; Shandong Public Health Clinical Center: Shandong Provincial Chest Hospital, CHINA

## Abstract

**Objective:**

Tuberculous meningitis (TBM) is one of the most devastating TB. Accurate identification of TBM is helpful to eliminate TB. Therefore, we assessed the performance of TBAg stimulated IFN-γ (IGRA) and unstimulated IFN-γ in blood and cerebrospinal fluid (CSF) for diagnosing TBM.

**Methods:**

We searched Web of Science, PubMed, Embase and the Cochrane Library databases until March 2022. Bivariate and hierarchical summary receiver operating characteristic models were employed to compute summary estimates for diagnostic accuracy parameters of IGRA and unstimulated IFN-γ in blood and CSF for diagnosing TBM.

**Results:**

28 studies including 1,978 participants and 2,641 samples met the inclusion criteria. The pooled sensitivity, specificity, positive likelihood ratio (PLR), negative likelihood ratio (NLR), diagnostic odds ratio (DOR) and area under the curve (AUROC) of blood IGRA were separately as 0.73, 0.83, 4.32, 0.33, 13.22 and 0.86, indicating a good diagnostic accuracy of blood IGRA for detecting TBM. The summary sensitivity, specificity, PLR, NLR, DOR and AUROC of CSF IGRA were separately as 0.77, 0.91, 8.82, 0.25, 34.59 and 0.93, indicating good diagnostic accuracy of CSF IGRA for detecting TBM. The summary sensitivity, specificity, PLR, NLR, DOR and AUROC of CSF IFN-γ were separately as 0.86, 0.92, 10.27, 0.16, 65.26 and 0.95, suggesting CSF IFN-γ provided excellent accuracy for diagnosing TBM.

**Conclusions:**

For differentiating TBM from non-TBM individuals, blood and CSF IGRA are good assays and unstimulated CSF IFN-γ is an auxiliary excellent marker.

## Introduction

Tuberculosis (TB) is one of the oldest infectious diseases and continues to threaten millions of citizens, especially in high-burden TB countries [1]. According to the 2020 global TB report, the World Health Organization (WHO) estimated that TB affected between 8.9 to 11.0 million individuals worldwide in 2019 [1]. Tuberculous meningitis (TBM) is one of the most devastating presentations of TB, with approximately 100,000 TBM cases reported annually [2, 3]. The nonspecific clinical features and delay in antitubercular therapy of TBM are responsible for neurological sequelae, seizures, and hydrocephalus, or even death in approximately 50% of patients [4, 5].

In defect of specific clinical symptoms and definite laboratory tests, early TBM diagnosis can be challenging. The composite diagnostic criteria of TBM include the examination of the presence of mycobacterium in the cerebrospinal fluid (CSF), histopathological, clinical, radiographical, and laboratory features [6]. Mycobacterial culture is the reference standard for the diagnosis of TBM, but its turn-around time of 6−8 weeks represents a significant limitation [5, 7]. Acid-fast bacilli (AFB) microscopy of stained CSF samples is the most widely used, rapid, and cost-effective diagnostic method; however, it has a poor sensitivity (10–15%) [8, 9]. Nucleic acid amplification tests (NAATs) are potential methods for TBM diagnosis, but they also have relatively low sensitivities (64% against CSF mycobacterial culture and 68% against a composite reference standard) [10, 11]. The CSF Xpert MTB/RIF test can rapidly differentiate TBM, with variable performance (sensitivity: 50–60%) and cost effectiveness [12]. Brain computed tomography (CT) and magnetic resonance imaging (MRI) are part of the clinical assessment of TBM, but they have limited clinical utility for early stage [4, 13]. Additionally, adenosine deaminase (ADA), the most commonly used laboratory parameter for TBM, has limited utility, especially in patients co-infected with the human immunodeficiency virus (HIV) [14]. Considering the limitations of the currently available methods, additional diagnostic assays for TBM are urgently needed.

Interferon-γ (IFN-γ) is a cytokine mainly produced and released by activated immune cells after *Mycobacterium*. *tuberculosis* infection [15, 16]. Moreover, IFN-γ is increased in the CSF of patients with TBM [17, 18]. IFN-γ includes *M*. *tuberculosis*-specific antigen (TBAg, like early secretary antigenic target 6, culture filtrate protein 10, etc) stimulated form and unstimulated form. Interferon-gamma release assay (IGRA), which is a TBAg-stimulated form of IFN-γ, detects the IFN-γ response of effector T cells to TBAg stimulation [3, 5]. Currently, IGRA can be performed by two approaches: QuantiFERON-TB Gold In-Tube (QFT-GIT) and T-SPOT.TB. In 2016, a meta-analysis of TBM yielded a summary sensitivity of 0.78 and specificity of 0.61 for blood IGRA, and a pooled sensitivity of 0.77 and specificity of 0.83 for CSF IGRA [19]. However, we found two studies that combined other central nervous system (CNS) TB (for example, intracranial tuberculoma) data along with the TBM data [20, 21]. In this study, we conducted a systematic review and meta-analysis to assess the diagnostic performance of TBAg-stimulated and unstimulated IFN-γ in in blood and CSF for detecting patients with TBM.

## Materials and methods

### Search strategy

Our team followed the 2018 guidelines from the Preferred Reporting Items for Systematic Reviews and Meta-Analyses Diagnostic Test Accuracy (PRISMA-DTA) statement published by McInnes et al [22]. This protocol was published on PROSPERO (CRD42021269819). We screened published reports stored in the Web of Science, PubMed, Embase, and the Cochrane Library databases since the inception of English citations indexed up to March 10, 2022. The following search terms were used: (tuberculous meningitis OR tuberculosis meningitis OR tubercular meningitis OR TBM OR meningeal tuberculosis OR extrapulmonary tuberculosis OR EPTB) AND (interferon OR IFN OR interferon-gamma OR gamma-interferon OR interferon-γ OR IFN-gamma OR IFN-γ OR interferon-gamma release assay OR IGRA OR QuantiFERON-TB OR QFT OR T-SPOT.TB). Furthermore, we reviewed the bibliographies of relevant articles to identify additional potential studies. An agreement of the Institutional Ethics Committee was not required because only publicly available reports were included in the analysis.

### Study selection

We selected original studies reporting IFN-γ for diagnosing TBM patients from non-TBM controls using the following criteria: (a) participants including TBM patients and non-TBM controls; (b) index test including two forms of IFN-γ, as TBAg-stimulated IFN-γ (IGRA, including QFT-GIT and T-SPOT.TB; TBAg, including early secretary antigenic target 6 and culture filtrate protein 10) and unstimulated IFN-γ in blood and CSF; (c) diagnostic standard for TBM including microbiologic in CSF (positivity of *M*. *tuberculosis* culture or NAAT or polymerase chain reaction, existence of AFB), histopathologic (presence of caseating granuloma in meninges), clinical, radiographic, and laboratory features (adequate resolution of hydrocephalus, granulomas, or basal exudates after empiric antitubercular therapy) [6]; (d) outcomes including sensitivity and specificity of IFN-γ, as well as those where the numerical data computed both these measures; (e) study designs, including randomized controlled trials and prospectively/retrospectively observational trials (cohort and cross-sectional studies). A publication including the largest participant samples was selected from among the identified articles reporting results on overlapping datasets. The optimal cutoff was selected among the articles reporting two or more diagnostic thresholds. The composite definition of the gold standard was adopted, which enrolled definite and probable TBM subjects.

We excluded all studies that were duplicated, non-English and animal publications, editorials and reviews, case reports, abstracts, biochemical and descriptive studies, studies without a control group, and articles presenting original data of less than five participants. Two investigators independently reviewed the titles and abstracts of the citations and full-text of potential studies to ensure that the inclusion criteria were fulfilled. Any disagreements were resolved by consensus.

### Data extraction and quality assessment

Two investigators independently extracted the following data from the eligible articles: first author name, date of publication, country where the study was conducted, TB burden, study design, number of participants (TBM/non-TBM individuals), diagnostic reference standard of TBM, and index test including the forms of assay (IGRA or unstimulated IFN-γ), method, cutoff, and TBAg-stimulated condition. In addition, according to different forms of IFN-γ (blood IGRA, CSF IGRA, unstimulated blood IFN-γ, and unstimulated CSF IFN-γ), blood and CSF samples, sensitivity, specificity, true positive (TP), false positive (FP), false negative (FN), and true negative (TN) data were extracted. The Quality Assessment of Diagnostic Accuracy Studies tool-2 (QUADAS-2), recommended by the Cochrane Collaboration, was used to summarize the methodological quality of the selected studies [23]. Deviations were resolved through consensus. The RevMan software (version 5.3; Cochrane, London, UK) was used to generate the figure of quality assessment.

### Statistical analysis

A bivariate random model was used to calculate the summary sensitivity, specificity, positive likelihood ratio (PLR), negative likelihood ratio (NLR), and diagnostic odds ratio (DOR) estimates for the different forms of IFN-γ, with 95% confidence intervals (CIs), as we expected significant heterogeneity in eligible studies. Furthermore, the area under the summary receiver operating characteristic curve (AUROC) was calculated. In general, AUROC below 0.75, between 0.75 and 0.93, or exceeding 0.93, indicated that the diagnostic accuracy of IFN-γ was not accurate, good, or excellent, respectively [24]. A hierarchical summary receiver operating characteristic (HSROC) model was constructed to summarize the overall diagnostic accuracy of IFN-γ [25]. We graphically identified the differences in the diagnostic performance across eligible studies using the HSROC curve.

*I*^2^ statistics was used to judge heterogeneity, with *I*^2^ > 0.75 indicating high heterogeneity [26]. The potential causes of heterogeneity were investigated using meta-regression and subgroup analyses. Subgroups were defined as different forms of TBAg-stimulated IFN-γ (T-SPOT.TB and QFT-GIT). Deeks’ funnel plot was used to graphically evaluate publication bias, in which *P* < 0.10 showed substantial asymmetry, suggesting the existence of bias [27]. All data were analyzed using the Stata software (version 14.0; StataCorp, College Station, TX, USA).

## Results

### Characteristics of the included articles

**Fig 1:**
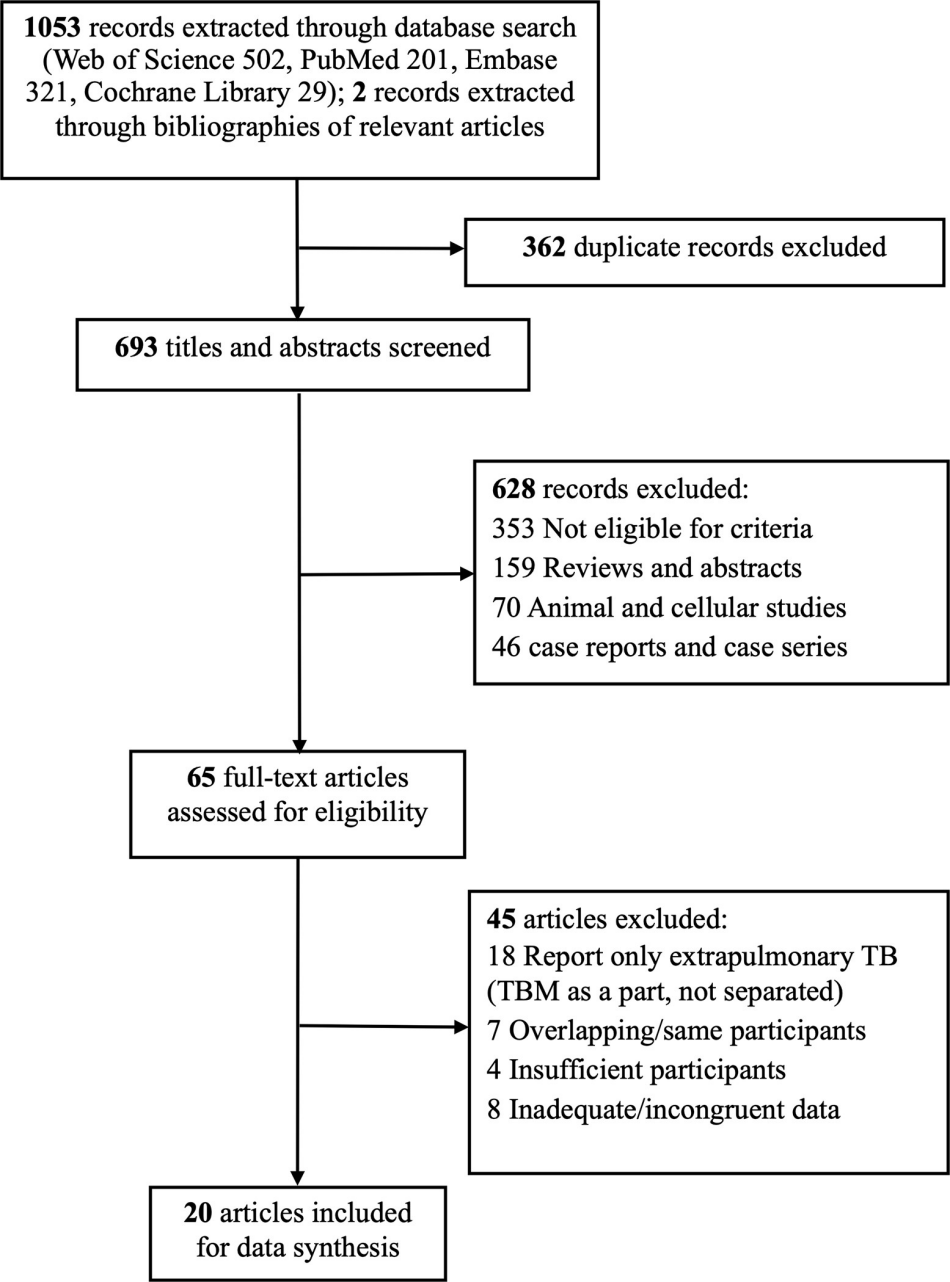
Articles’ selection process.

We identified 1053 records across four electronic databases and 2 records from bibliographies of relevant articles, among which 362 duplicated records were excluded. Following an initial screening of titles and abstracts, we excluded 628 records, of which 353 records were not eligible as it included other diseases (latent tuberculosis infection, cryptococcal meningitis, chronic hepatitis B, among others) and other index tests (NAAT, Xpert MTB/RIF assay, neutrophil CD64, among others); 159 records were non-eligible types of reports including reviews, recommendations, consensus, systematic review/meta-analysis, and abstracts; 70 records were of animal and cellular studies; and 46 records were case reports and case series. We then assessed 65 full-text articles in detail (Fig 1). Ultimately, 20 articles were selected for characteristic collection and data analysis (Tables 1 and 2) [28–47].

**Table 1:** Characteristics of the included articles.

Author	Year	Country	TB-burden	Study design	TBM/non-TBM individuals (N)	Reference standard	Index test			
							Assay	Method	Cut-off	TBAg stimulated
Luo Y [28]	2021	China	High	Prospectively	76/98	Composite	IGRA (T-SPOT.TB)	ELISPOT	6 SFCs (Blood)	Yes
Luo Y [28]	2021	China	High	Prospectively	39/66	Composite	IGRA (T-SPOT.TB)	ELISPOT	6 SFCs (Blood)	Yes
Kwon JS [29]	2019	Republic of Korea	Low	Prospectively	10/45	Composite	IGRA (T-SPOT.TB)	ELISPOT	14 SFCs (Blood)/13.5 SFCs (CSF)	Yes
Manyelo CMa [30]	2019	South Africa	High	Prospectively	23/24	Composite	IFN-γ	Luminex kits	1.00 IU/ml (CSF)	No
Manyelo CMb [31]	2019	South Africa	High	Prospectively	23/24	Composite	IFN-γ	Luminex kits	0.62 IU /ml (Blood)	No
Di L [32]	2018	China	High	Retrospectively	8/251	Composite	IGRA (T-SPOT.TB)	ELISPOT	6 SFCs (Blood)	Yes
Li XL [33]	2017	China	High	Retrospectively	52/44	Composite	IGRA (T-SPOT.TB)	ELISPOT	10 SFCs (CSF)	Yes
Pan L [34]	2017	China	High	Prospectively	53/37	Composite	IGRA (T-SPOT.TB)	ELISPOT	24 SFCs (Blood)/20 SFCs (CSF)	Yes
Lu T [35]	2017	China	High	Retrospectively	43/85	Composite	IGRA (T-SPOT.TB)	ELISPOT	6 SFCs (Blood)	Yes
Park KH [36]	2016	Republic of Korea	Low	Prospectively	49/186	Composite	IGRA (T-SPOT.TB)	ELISPOT	45 SFCs (Blood)/12 SFCs (CSF)	Yes
Lu D [37]	2016	China	High	un	30/39	Composite	IGRA (T-SPOT.TB)	ELISPOT	6 SFCs (Blood)	Yes
Lu D [37]	2016	China	High	un	30/39	Composite	IFN-γ	ELISA	0.81 IU /ml (CSF)	No
El Azbaoui S [38]	2016	Morocco	Low	Prospectively	12/28	Composite	IGRA (QFT-GIT)	ELISA	0.35 IU/ml (Blood)	Yes
Qin L [39]	2015	China	High	Prospectively	12/28	Composite	IGRA (T-SPOT.TB)	ELISPOT	6 SFCs (Blood/CSF)	Yes
Caliman-Sturdza OA [40]	2015	Romania	Low	un	63/62	Composite	IGRA (QFT-GIT)	ELISA	0.35 IU/ml (Blood/CSF)	Yes
Vidhate MR [41]	2011	India	High	un	24/16	Composite	IGRA (QFT-GIT)	ELISA	10 IU/ml (Blood)	Yes
Patel VB [42]	2011	South Africa	High	Prospectively	37/15	Composite	IFN-γ	ELISA	0.244 IU/ml (CSF)	No
Patel VB [43]	2010	South Africa	High	Prospectively	94/48	Composite	IGRA (T-SPOT.TB)	ELISPOT	46 SFCs (CSF)	Yes
Liao CH [44]	2009	Taiwan	Low	Prospectively	6/14	Composite	IGRA (T-SPOT.TB)	ELISPOT	10 SFCs (Blood)	Yes
Chen X [45]	2009	China	High	Prospectively	16/121	Composite	IGRA (T-SPOT.TB)	ELISPOT	11 SFCs (Blood)	Yes
Thomas MM [46]	2008	India	High	Prospectively	11/9	Composite	IGRA (T-SPOT.TB)	ELISPOT	5 SFCs (Blood/CSF)	Yes
San Juan R [47]	2006	Spain	Low	Prospectively/Retrospectively	20/37	Composite	IFN-γ	Radioimmunoassay	6.4 IU/ml (CSF)	No

Abbreviations: TB: tuberculosis; TBM: tuberculous meningitis; IFN-γ: interferon; IGRA: interferon-gamma release assay; QFT-GIT: QuantiFERON-TB Gold in-tube; ELISA: enzyme-linked immuno sorbent assay; ELISPOT: enzyme-linked immuno spot assay; SFCs: spot-forming cells; CSF: cerebrospinal fluid; IU: international unit; TBAg: M. tuberculosis-specific antigen.

**Table 2:** Diagnostic performance of IFN-γ in the individual studies.

Author	Year	Samples		Sensitivity% (95% CI)	Specificity% (95% CI)	TP	FP	FN	TN
		TBM	non-TBM						
**Blood IGRA (T-SPOT.TB and QFT-GIT)**									
Luo Y	2021	76	98	85.53	81.63	65	18	11	80
Luo Y	2021	39	66	79.49	77.27	31	15	8	51
Kwon JS	2019	10	45	50.0 (18.7–81.3)	77.8 (62.9–88.8)	5	10	5	35
Di L	2018	8	251	25	92.83	2	18	6	233
Pan L	2017	53	37	90.6 (79.3–96.9)	75.7 (58.8–88.2)	48	9	5	28
Lu T	2017	43	85	72.1 (56.1–84.2)	72.9 (62.0–81.7)	31	23	12	62
Park KH	2016	46	159	65 (50–79)	84 (78–90)	30	25	16	134
Lu D	2016	30	39	70 (54–86)	87 (73–96)	21	5	9	34
Qin L	2015	12	28	83 (52–98)	82 (63–94)	10	5	2	23
Liao CH	2009	6	14	100	71.4	6	4	0	10
Chen X	2009	16	121	62.5	85.95	10	17	6	104
Thomas MM	2008	11	8	82 (48–98)	75 (35–97)	9	2	2	6
El Azbaoui S	2016	10	28	80	100	8	0	2	28
Caliman-Sturdza OA	2015	58	58	77.6	87.9	45	7	13	51
Vidhate MR	2011	24	16	33.3	62.5	8	6	16	10
**CSF IGRA (T-SPOT.TB and QFT-GIT)**									
Kwon JS	2019	10	45	30.0 (6.7–65.3)	91.1 (78.8–97.5)	3	4	7	41
Li XL	2017	52	44	97.8 (87–99.9)	78 (63.7–88)	51	10	1	34
Pan L	2017	51	36	60.8 (46.1–74.2)	97.2 (85.5–99.9)	31	1	20	35
Park KH	2016	38	109	66 (49–80)	90 (83–95)	25	11	13	98
Qin L	2015	12	28	92 (62–100)	93 (76–99)	11	2	1	26
Patel VB	2010	92	48	52 (42–63)	83 (70–93)	48	8	44	40
Thomas MM	2008	10	7	90 (56–100)	100 (59–100)	9	0	1	7
Caliman-Sturdza OA	2015	56	56	80.4	98.2	45	1	11	55
**Blood unstimulated IFN-γ**									
Manyelo CMb	2019	23	24	87.0 (66.4–92.2)	20.8 (7.1–42.2)	20	19	3	5
**CSF unstimulated IFN-γ**									
Manyelo CMa	2019	23	24	91.3 (72.0–98.9)	91.7 (73.0–99.0)	21	2	2	22
Lu D	2016	30	39	83 (65–94)	85 (69–93)	25	6	5	33
Patel VB	2011	37	15	92 (78–98)	100 (78–100)	34	0	3	15
San Juan R	2006	20	37	70 (50–90)	95 (90–98)	14	2	6	35

Abbreviations: IFN-γ: interferon; IGRA: interferon-gamma release assay; QFT-GIT: QuantiFERON-TB Gold in-tube; TBM: tuberculous meningitis; CSF: cerebrospinal fluid; CI: Confidence interval; TP: true-positive; FP: false-positive; FN: false-negative; TN: true-negative

The final dataset of 20 articles comprised 1,978 participants (701 TBM patients and 1,277 non-TBM individuals), with an overall TBM prevalence of 35.44%. Fourteen (70%) articles were reported from countries recognized as ‘high burden’ by the WHO [28, 30–35, 37, 39, 41–43, 45, 46]. Thirteen publications (65%) were of prospective studies [28–31, 34, 36, 38, 39, 42–46]. All the studies used various composite reference standards. Four studies estimated unstimulated CSF IFN-γ levels with different methods (Luminex kits, ELISA, radioimmunoassay) and diagnostic thresholds (0.244–6.4 IU/mL) [30, 37, 42, 47], and one study tested unstimulated blood IFN-γ levels using Luminex kits assay with a cutoff of 0.62 IU/mL [31]. Twenty-three studies used IGRA (T-SPOT.TB and QFT-GIT) to estimate TBAg-stimulated IFN-γ levels using ELISPOT and ELISA methods, including eight studies that analyzed CSF samples and fifteen studies that analyzed blood samples. Diagnostic thresholds of the T-SPOT.TB differed widely from to 5–46 spot-forming cells (SFCs) in CSF and 5–45 SFCs in blood, with six being the most used manufacturers’ recommended cutoff. According to the manufacturer’s recommendation, the cutoff value of QFT-GIT was 0.35 IU/mL in CSF and blood, while one study used 10 IU/mL in blood (Table 1). In total, 28 studies included 2,641 samples (896 TBM samples and 1,565 non-TBM samples) from blood and CSF. Diagnostic accuracy estimates of blood and CSF IGRA (T-SPOT.TB and QFT-GIT) and unstimulated IFN-γ-detecting TBM are shown in Table 2.

### Quality of the eligible articles

**Fig 2:**
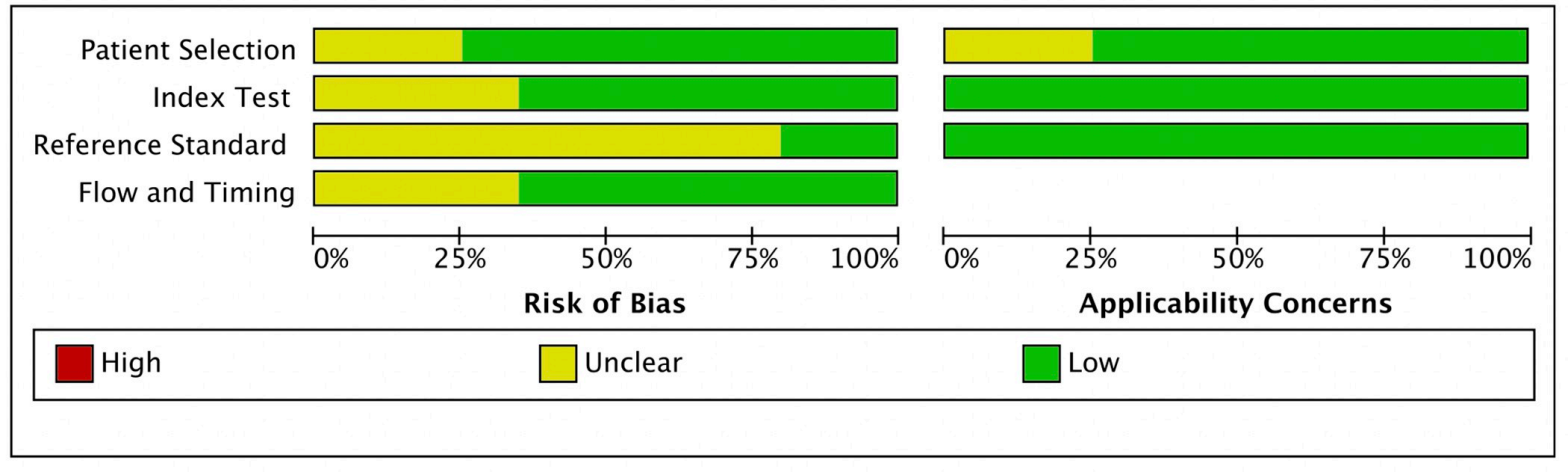
Quality for the included articles.

According to the QUADAS-2 tool, we found a low risk of quality bias for the included articles (Fig 2). Patient selection bias was unclear for five articles (5/20, 25%), as investigators did not report consecutive selection for patient included criteria [33, 40, 44, 45, 47]. The unclear bias primarily resulted in the index test domain (7/20, 35%) [32, 33, 37, 38, 40, 41, 44] and reference standard domain (16/20, 70%) [28–33, 35–41, 44, 45, 47], as reviewers failed to judge study blinding by insufficient information. Flow and timing bias were unclear for seven articles (7/20, 35%), as investigators excluded intermediate results of IFN-γ and/or missing data of participants [29, 34, 36, 38, 40, 43, 46]. Five (5/20, 25%) studies additionally had unclear applicability concerns in the patient selection domain, as the main characteristics of TBM were unclear, in which these publications focused on extrapulmonary TB [32, 33, 38, 44, 45].

### Diagnostic accuracy of blood IGRA

**Fig 3:**
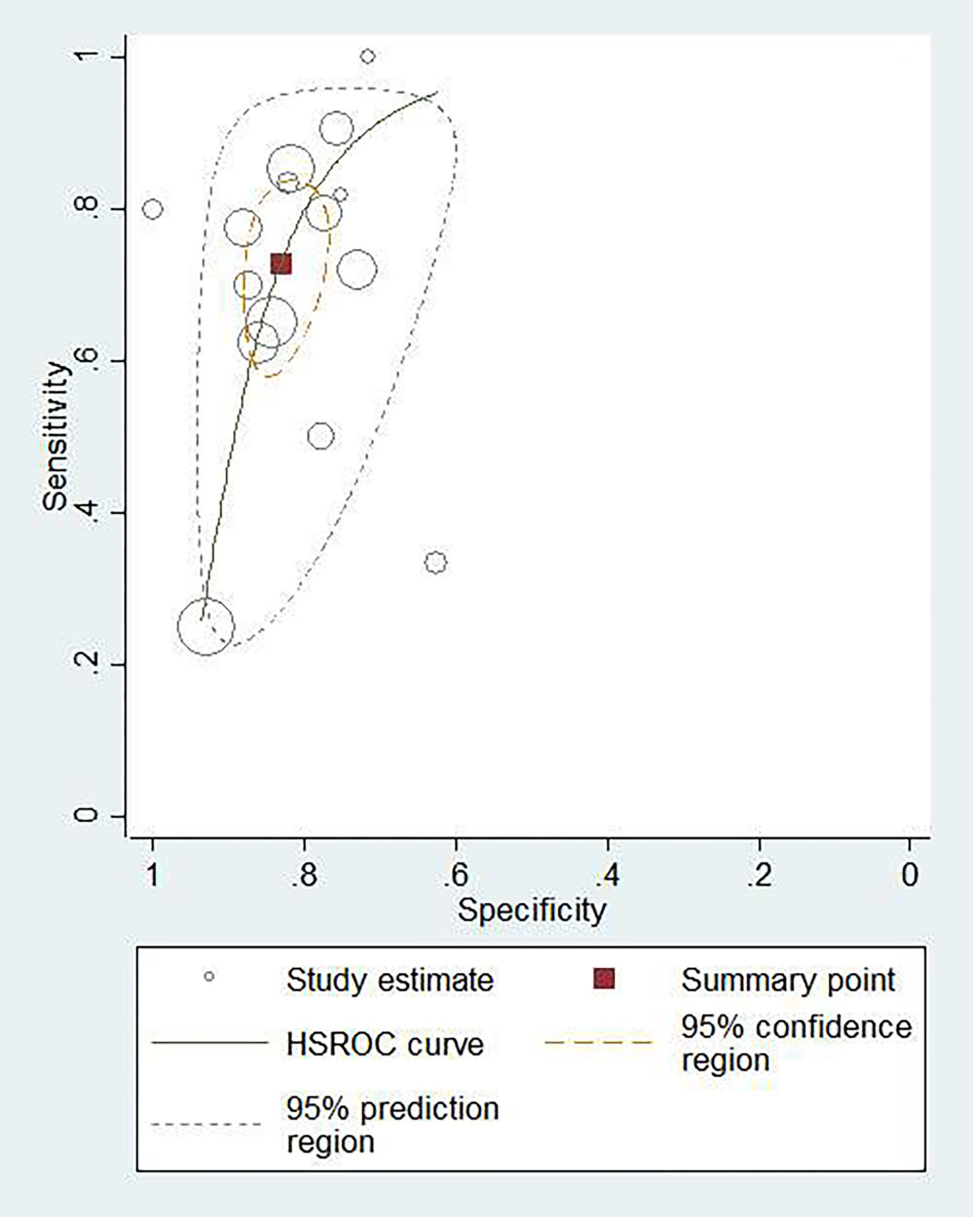
Hierarchical summary receiver operating characteristic (HSROC) plot to summarize diagnostic accuracy for blood IGRA in diagnosing tuberculous meningitis (blue curve). Summary estimate of diagnostic accuracy is indicated by the red square.

To differentiate TBM individuals from non-TBM controls, a total of 1,495 blood samples from 15 studies using IGRA were evaluated. The sensitivity ranged from 0.25 to 1.00, with a summary sensitivity of 0.73 (95% CI: 0.63–0.81, *I*^2^: 74.70%) (S1 Fig). The specificity varied between 0.63 and 1.00, with a summary specificity of 0.83 (95% CI: 0.79–0.87, *I*^2^: 66.68%) (S1 Fig). The summary PLR and NLR were 4.32 (95% CI: 3.42–5.46) and 0.33 (95% CI: 0.23–0.46), respectively. The summary DOR was 13.22 (95% CI: 8.22–21.25). The AUROC was 0.86 (95% CI: 0.83–0.89), suggesting that the diagnostic accuracy of blood IGRA was good. The HSROC curve of blood IGRA is shown in Fig 3.

We observed relatively high heterogeneity (*I*^*2*^ = 74.70% for sensitivity) among the eligible studies. In the meta-regression and subgroup analyses, the different forms of blood TBAg stimulated IFN-γ (T-SPOT.TB and QFT-GIT) was not the cause of heterogeneity (*P* = 0.46). The pooled sensitivity of blood T-SPOT.TB was higher when compared with QFT-GIT (0.75 *vs*. 0.64). The pooled specificity of T-SPOT.TB was lower when compared with QFT-GIT (0.82 *vs*. 0.88).

### Diagnostic accuracy of CSF IGRA

**Fig 4:**
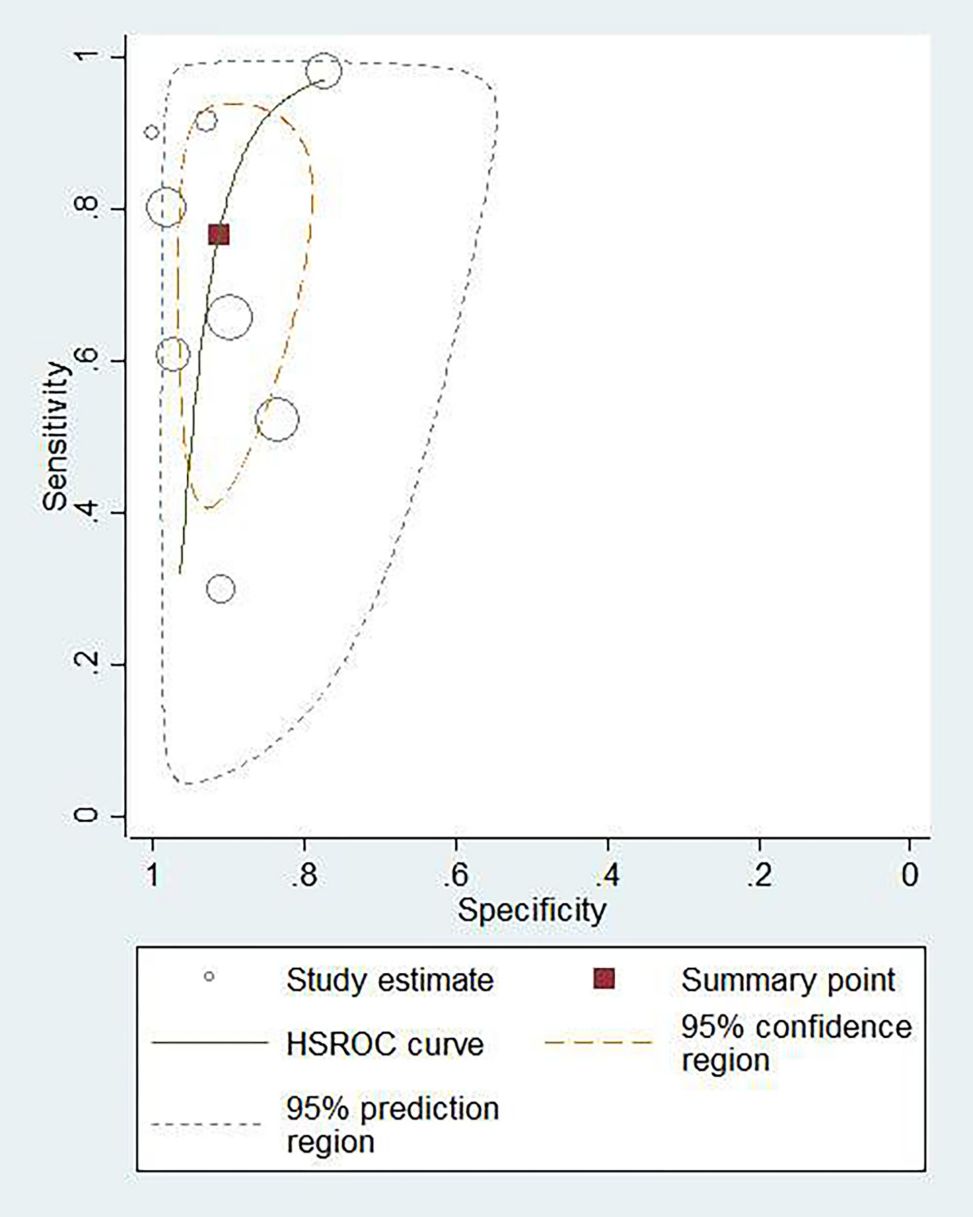
Hierarchical summary receiver operating characteristic (HSROC) plot to summarize diagnostic accuracy for CSF IGRA in diagnosing tuberculous meningitis.

Eight studies, comprising 694 CSF samples, were included in the IGRA group. The summary sensitivity and specificity were 0.77 (95% CI: 0.56–0.90; *I*^*2*^: 87.76%) and 0.91 (95% CI: 0.85–0.95; *I*^*2*^: 68.04%), respectively (S2 Fig). The summary PLR and NLR were 8.82 (95% CI: 4.97–15.63) and 0.25 (95% CI: 0.12–0.53), respectively. The summary DOR was 34.59 (95% CI: 12.25–97.64). The AUROC was 0.93 (95% CI: 0.91–0.95), suggesting that the diagnostic accuracy of CSF IGRA was good. The HSROC curve of CSF IGRA is shown in Fig 4.

High heterogeneity was observed between the included studies (*I*^*2*^ = 87.76% sensitivity). In the meta-regression and subgroup analyses, the different forms of CSF TBAg stimulated IFN-γ (T-SPOT.TB and QFT-GIT) might not be the cause of heterogeneity (*P* = 0.07). The pooled sensitivity and specificity of CSF T-SPOT.TB were 0.75 and 0.88, respectively. In addition, the pooled sensitivity and specificity of CSF QFT-GIT were 0.81 and 0.98, respectively.

### Diagnostic accuracy of unstimulated blood and CSF IFN-γ

**Fig 5:**
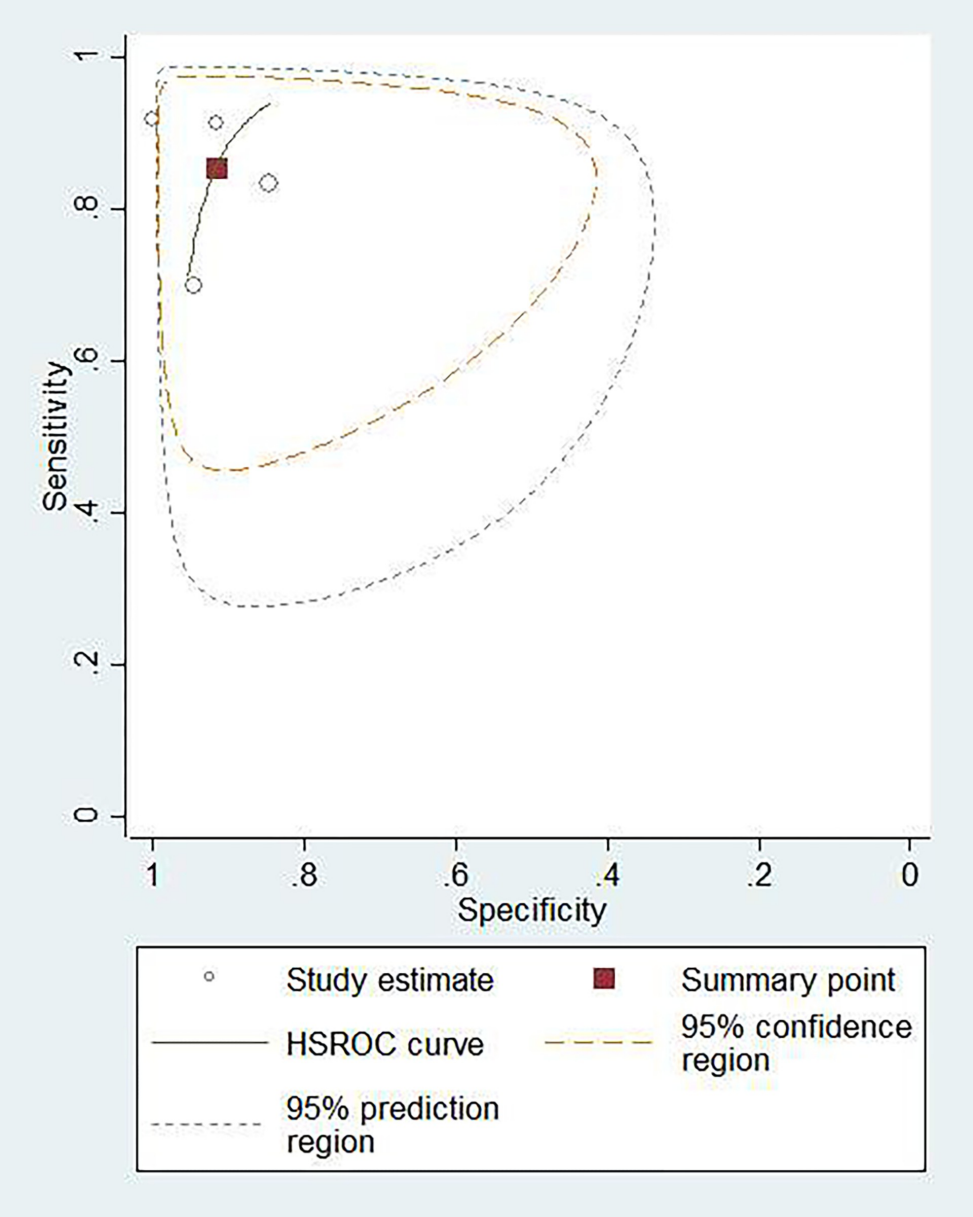
Hierarchical summary receiver operating characteristic (HSROC) plot to summarize diagnostic accuracy for unstimulated CSF IFN-γ in diagnosing tuberculous meningitis.

One study reported a low specificity (0.21) for unstimulated blood IFN-γ. Four studies, including 225 CSF samples, were performed using unstimulated IFN-γ. The summary sensitivity and specificity of 0.86 (95% CI: 0.76–0.92; *I*^*2*^: 48.70%) and 0.92 (95% CI: 0.82–0.96; *I*^*2*^: 31.75%), respectively (S3 Fig). The summary PLR and NLR were 10.27 (95% CI: 4.57–23.08) and 0.16 (95% CI: 0.09–0.27), respectively. The summary DOR was 65.26 (95% CI: 21.69–196.34). The AUROC was 0.95 (95% CI: 0.93–0.97), suggesting that the diagnostic accuracy of unstimulated CSF IFN-γ was excellent. The summary point was located near the preferred upper left corner of the HSROC curve, indicating that unstimulated CSF IFN-γ was a good discriminator (Fig 5).

### Publication bias

Deeks’ funnel plots showed no statistically significant differences in blood IGRA (*P* = 0.61), CSF IGRA *(P =* 0.84), and unstimulated CSF IFN-γ (*P* = 0.12). Therefore, there was no publication bias in this study.

## Discussion

Our results suggest that blood IGRA demonstrates good diagnostic potential in TBM diagnosis with an AUROC of 0.86. CSF IGRA also showed good diagnostic performance for TBM diagnosis with an AUROC of 0.93. Further, our analysis revealed that the different types of IGRA was not the cause of heterogeneity. In the case of active TB, *M*. *tuberculosis*-specific effector T cells could be recruited to the infection site, and then IGRA was used to detect TBAg-stimulated IFN-γ at the infection site (for example, using CSF), which could present a higher specificity of TB diagnosis compared with blood IGRA [20, 32, 48]. Our study revealed a higher diagnostic specificity (0.91) for CSF IGRA; however, the specificity of blood IGRA was of 0.83. Our study also showed a slightly higher sensitivity (0.77) of CSF IGRA according to data from eight studies, whereas the sensitivity of blood IGRA was 0.73 based on 15 studies. We assume that the possible reason for lower sensitivity is the protective effect of the blood-brain barrier [32].

Using eight original articles, Yu et al. (2016) performed a meta-analysis of blood and CSF IGRA for distinguishing between TBM and non-TBM individuals [19], in which blood IGRA had a pooled sensitivity of 0.78 and specificity of 0.61, whereas CSF IGRA had a pooled sensitivity of 0.77 and specificity of 0.83. However, this analysis included one article that combined other CNS TB (such as intracranial tuberculoma) patient data into the TBM data [20]. Moreover, three other articles had overlapping datasets [21, 48, 49]. Therefore, a 2016 new research including the largest participant samples was selected for our study. In addition, the remaining four original articles from the meta-analysis by Yu et al. were selected. We have improved the reliability and stability of the diagnostic performance of IGRA for detecting TBM by analyzing 14 additional publications. Furthermore, we analyzed the unstimulated IFN-γ.

In 2020, Luo et al. included 27 articles and showed that, for TBM diagnosis, the pooled sensitivity and specificity of blood T-SPOT.TB were 0.78 and 0.68, and the pooled sensitivity and specificity of CSF T-SPOT.TB were 0.76 and 0.88 [50]. However, this research also included intracranial tuberculoma patient data for one article [20] and overlapping datasets in four articles [21, 36, 48, 49], causing unreliable pooled results. Besides, 14 eligible articles are in Chinese, which might lead to biased results. Our study included a newest article with the largest participant samples [36], excluding overlapping datasets [21, 48, 49] and admixture of other brain tuberculosis [20], which conducted more truthful results. Furthermore, we included other 11 original articles and analyzed the performance of QFT-GIT and unstimulated IFN-γ for TBM. In 2021, Lan et al. included 26 publications, and found blood IGRA had a pooled sensitivity of 0.81 and specificity of 0.76 for TBM, and CSF IGRA had a pooled sensitivity of 0.81 and specificity of 0.89 [51]. This meta-analysis had the same problems as Luo et al., in which three included articles had overlapping datasets [21, 36, 49] and 17 were in Chinese. Our study excluded overlapping datasets [21, 49] and Chinese publications, which could increase the reliability of pooled results. Besides, we included other 12 original articles and estimated the detective value of unstimulated IFN-γ.

A systematic review published by Aggarwal et al. in 2021, comprising 7,153 patients, showed that the IFN-γ of unstimulated pleural fluid provided excellent accuracy for diagnosing tuberculous pleural effusion (sensitivity: 0.93, specificity: 0.96) [52]. To date, the diagnostic performance of unstimulated CSF IFN-γ for TBM remained unclear. In our study, unstimulated CSF IFN-γ had a relatively high summary sensitivity (0.86) and specificity (0.92). The AUROC was of 0.95, suggesting that the diagnostic accuracy of unstimulated CSF IFN-γ was excellent. In 2017, Pormohammad et al. performed a systematic review of CSF ADA, indicating that CSF ADA had a relatively high accuracy for TBM diagnosis (sensitivity, 0.89; specificity, 0.91; AUROC, 0.96) [53]. The diagnostic accuracy estimates of the unstimulated CSF IFN-γ in the current study are similar to those reported for CSF ADA by Pormohammad et al. Overall, this suggests that unstimulated CSF IFN-γ may be as useful as currently common diagnostic biomarkers, such as ADA, for TBM.

Our meta-analysis had several limitations. First, we could not rule out a misclassification bias because all studies used composite criteria for diagnosing TBM. Second, the different forms of TBAg-stimulated IFN-γ were not the significant source of heterogeneity, however, we did not evaluate other possible factors (such as patient age, HIV-coinfection condition). Furthermore, for TBAg-unstimulated IFN-γ, we did not analyze potential factors for heterogeneity, e.g., clinical types of non-TBM participants, which might influence the interpretability of the pooled results. Third, some studies derived diagnostic thresholds of stimulated IFN-γ in a post-hoc fashion by optimizing the trade-off between specificity and sensitivity, rather than using a manufacturer recommended cutoff, which could be more clinically appropriate for confirmation or exclusion of a TBM diagnosis. Additionally, there was no publication bias in this study; however, we only included studies published in English journals; thus, a concern for publication bias could not be excluded. In clinic, a large CSF sample was needed for the stimulation of IFN-γ among TBM patients with few lymphocytes in the CSF, which is a challenge. Moreover, different age may affect the diagnosis of TBM. In further study, for TBM detection, it is better to explore the pediatric cases and adult patients separately.

In conclusion, this systematic review and meta-analysis shows that blood and CSF IGRA are good assays for differentiating TBM from non-TBM individuals, and unstimulated CSF IFN-γ is an auxiliary excellent marker for detecting TBM. Further large-scale, multi-center, prospective studies are warranted to support our findings, especially the findings on unstimulated CSF IFN-γ.

## Supporting information

S1 Checklist PRISMA 2020 checklist. (DOCX)

S1 Fig The pooled sensitivity and specificity of blood IGRA for diagnosing tuberculous meningitis. (TIF)

S2 Fig The pooled sensitivity and specificity of CSF IGRA for diagnosing tuberculous meningitis. (TIF)

S3 Fig The pooled sensitivity and specificity of unstimulated CSF IFN-γ for diagnosing tuberculous meningitis. (TIF)
