# Supplementary figures and images for: Tuberculosis-specific antigen stimulated and unstimulated interferon-γ for tuberculous meningitis diagnosis: A systematic review and meta-analysis
Source: PLoS One. 2022 Aug 30;17(8):e0273834. doi: 10.1371/journal.pone.0273834 (PMC9426936; doi:10.1371/journal.pone.0273834)

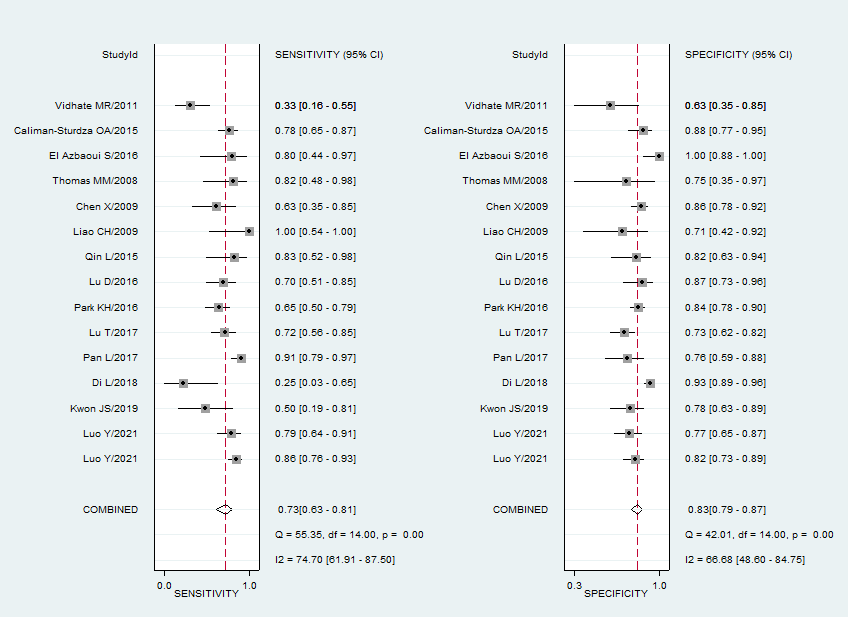

Supplement: S1 Fig — (TIF) [file pone.0273834.s002.tif]

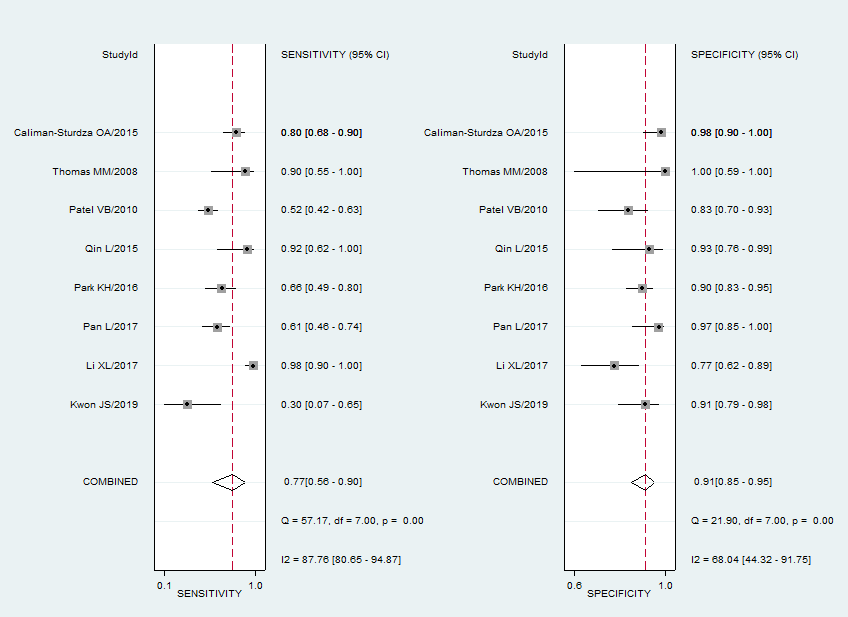

Supplement: S2 Fig — (TIF) [file pone.0273834.s003.tif]

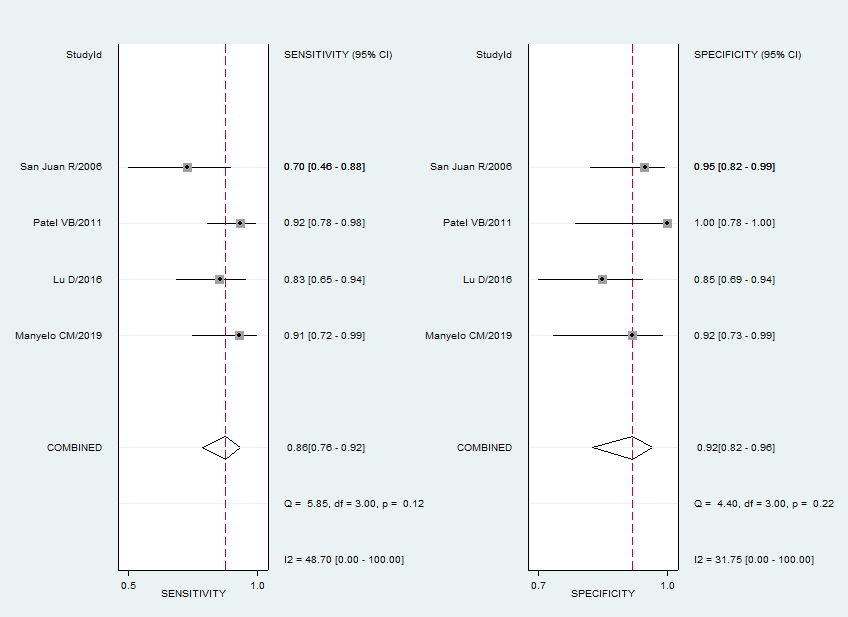

Supplement: S3 Fig — (TIF) [file pone.0273834.s004.tif]
